# Characteristics of the Microbial Community in the Production of Chinese Rice-Flavor Baijiu and Comparisons With the Microflora of Other Flavors of Baijiu

**DOI:** 10.3389/fmicb.2021.673670

**Published:** 2021-04-29

**Authors:** Yuanliang Hu, Xinyi Lei, Xiaomin Zhang, Tongwei Guan, Luyao Wang, Zongjie Zhang, Xiang Yu, Junming Tu, Nan Peng, Yunxiang Liang, Shumiao Zhao

**Affiliations:** ^1^Hubei Key Laboratory of Edible Wild Plants Conservation and Utilization, College of Life Sciences, Hubei Normal University, Huangshi, China; ^2^State Key Laboratory of Agricultural Microbiology, College of Life Science and Technology, Huazhong Agricultural University, Wuhan, China; ^3^Hubei Engineering Research Center of Typical Wild Vegetable Breeding and Comprehensive Utilization Technology, Huangshi, China; ^4^Guangdong Deqing Incomparable Health Wine Co., Ltd., Zhaoqing, China; ^5^College of Food and Biological Engineering, Xihua University, Chengdu, China

**Keywords:** baijiu, fungal community, bacterial community, high-throughput sequencing, baijiu flavor

## Abstract

Rice-flavor baijiu is one of the four basic flavor types of Chinese baijiu. Microbial composition plays a key role in the classification of baijiu flavor types and the formation of flavor substances. In this study, we used high-throughput sequencing technology to study the changes of microbial community in the production of rice-flavor baijiu, and compared the microbial community characteristics during production of rice-, light-, and strong-flavor baijiu. The results showed that the species diversity of bacteria was much higher than that of fungi during the brewing of rice-flavor baijiu. The bacterial diversity index first increased and then decreased, while the diversity of fungi showed an increasing trend. A variety of major microorganisms came from the environment and raw rice materials; the core bacteria were *Lactobacillus*, *Weissella*, *Pediococcus*, *Lactococcus*, *Acetobacter*, etc., among which *Lactobacillus* was dominant (62.88–99.23%). The core fungi were *Saccharomyces* (7.06–83.50%) and *Rhizopus* (15.21–90.89%). Temperature and total acid content were the main physicochemical factors affecting the microbial composition. Non-metric multidimensional scaling analysis showed that during the fermentation of rice-, light-, and strong-flavor baijiu, their microbial communities formed their own distinct systems, with considerable differences among different flavor types. Compared with the other two flavor types of baijiu, in the brewing process of rice-flavor baijiu, microbial species were fewer and dominant microorganisms were prominent, which may be the main reason for the small variety of flavor substances in rice-flavor baijiu. This study provides a theoretical basis for the production of rice-flavor baijiu, and lays a foundation for studying the link between baijiu flavor formation and microorganisms.

## Introduction

Chinese baijiu is a traditional alcoholic beverage and one of the six major distilled spirits in the world (Wu J. et al., 2017; He et al., 2019). Compared with other distilled spirits, such as whisky and brandy, baijiu is generally produced by natural solid-state fermentation with a mixture of microbial species (yeasts, bacteria, and molds), with saccharification and fermentation taking place at the same time, and the base liquor being obtained by solid-state distillation. In contrast, other distilled spirits undergo saccharification and fermentation separately, enzyme preparation is added for saccharification, one or more yeasts are inoculated for liquid fermentation, and the base liquor is obtained by liquid distillation (Jin et al., 2017; Wanikawa, 2020). Due to the differences in the selection of raw materials, types of distillers, and production processes, etc., 12 types of baijiu have been developed in China, with four main flavor types; sauce, strong, light, and rice flavors (Yin et al., 2020a; Gao et al., 2021).

**FIGURE 1:**
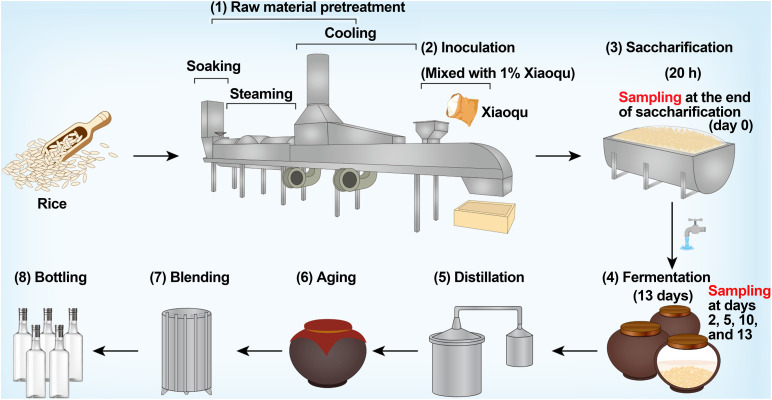
Production process of rice-flavor baijiu and sampling design in this study.

Rice-flavor baijiu is a rare type of baijiu in China that is brewed from rice (Yin et al., 2020a). The production process mainly includes raw material pretreatment, inoculation, saccharification, fermentation, distillation, aging, blending, and bottling (Figure 1). The microbial starter of rice-flavor baijiu is Xiaoqu, which provides various functional microorganisms, hydrolases, volatile substances, and other components for baijiu brewing, forming the unique flavor of baijiu (Arbab et al., 2020; Wang et al., 2021). During saccharification, the microorganisms and enzymes in Xiaoqu degrade the starch in rice into sugar (Wang J. et al., 2018). At the end of saccharification, water for brewing is added, semi-solid fermentation is carried out for approximately 13 days, and the base liquor is finally obtained by distillation (Li et al., 2019).

The essence of baijiu-making is the process of microbial growth and accumulation of metabolites, and the synergistic effect among populations is closely related to the flavor and quality of baijiu (He et al., 2019). *Saccharomyces* can ferment sugars to produce alcohol, and most of them can survive under high-sugar and relatively acidic conditions and enable alcoholization and esterification during the fermentation of baijiu (Liu and Miao, 2020). Among them, *Saccharomyces cerevisiae* mainly plays an alcohol-producing role and has a strong alcohol-producing capacity (Wang et al., 2020). Most of the other yeasts are ester-producing yeasts, and although their alcohol production volume is relatively small, they can convert production materials into aldehydes, esters, higher alcohols, etc., during baijiu fermentation. Molds can produce hydrolases, such as amylase, protease, and lipase, for the saccharification of starch and breakdown of macromolecules, and these enzymes provide important compounds that contribute to the formation of flavor (Wang B. et al., 2018). Bacteria are mainly used to produce flavor components and precursors of flavor components, which are important sources of the unique flavor of baijiu (Dai et al., 2020). The synergistic action of many microorganisms completes the baijiu brewing process and creates the unique style of baijiu.

Rice-flavor baijiu is very different from the other three major flavor types of baijiu; light-, strong-, and sauce-flavor (Yin et al., 2020a). These three flavor types are all spicy, while the rice-flavor type has a sweet taste, a honey aroma, a quietly elegant, and a soft mouth feel. The “rice-brewing aroma” and “Xiaoqu baijiu aroma” comprise the elegant and soft aroma composed of three substances, namely, ethyl lactate, ethyl acetate, and β-phenylethanol. Microorganisms play a decisive role in the formation of flavor substances and flavor types of baijiu (Wang et al., 2019; Huang et al., 2020). Lactic acid and acetic acid are the precursors of ethyl lactate and ethyl acetate, respectively. Lactic acid is mainly derived from *Lactobacillus* spp., while acetic acid is mainly produced by the oxidation of ethanol by *Acetobacter* spp. In addition, *Rhizopus* produces glucoamylase and proteases as well as important flavor substances, such as lactic acid, other organic acids, and aromatic series. Yeasts can produce aldehyde esters, β-phenylethanol, and other higher alcohols during fermentation, and form flavor substances with ethyl acetate as the main ester (Guan et al., 2020). Among the many microbial groups, only the core microorganisms can drive the fermentation process, and they not only produce various flavor substances, but more importantly, they can maintain the interactions among microorganisms, which together determine the quality of baijiu (Song et al., 2017). Therefore, the study of microbial composition and diversity is of great significance for gaining insight into the style characteristics and brewing mechanisms of Chinese baijiu.

Both rice-flavor baijiu and Xiaoqu light-flavor baijiu use Xiaoqu as the microbial starter and have very similar brewing cycles, yet they are different flavor types of baijiu with their own styles. It is currently unclear whether there are differences in the composition of the microbial communities in the production of these two flavor types of baijiu. In addition, there have been more studies of the brewing microorganisms of light- (Dong et al., 2020; Hu et al., 2021b), strong- (Guan et al., 2020), and sauce-flavor baijiu (Dai et al., 2020), but less of rice-flavor baijiu, and its brewing microbial composition has yet to be resolved. Therefore, in this study, high-throughput sequencing technology was used to analyze the microbial communities in the starters and production processes of rice-flavor baijiu to identify the core microbiota and study their effects on the microbial communities by combining with changes in physicochemical factors, such as flavor components. In addition, high-throughput sequencing and bioinformatics analysis were used to compare the microbial community composition of rice-, light-, and strong-flavor baijiu brewing, laying a foundation for an in-depth study of the nature of baijiu flavor formation and the linkage among microorganisms.

## Materials and Methods

### Brewing Process and Sampling

The production process of rice-flavor baijiu is shown in Figure 1. Fermentation occurred in clay vats sealed for 13 days after 20 h of saccharification. Both saccharification and fermentation took place at room temperature. Samples were taken at the end of saccharification (d0) and at the end of fermentation on days 2 (d2), 5 (d5), 10 (d10), and 13 (d13). Three replicates were sampled each time, i.e., from three different vats. A five-point sampling method was used to reduce sampling errors, i.e., five sub-samples were mixed into one test sample with the sampling point being 30 cm below the surface of the fermented rice, and the sampling volume each time was approximately 600 g. After mixing well, 50 g of samples were snap frozen in liquid nitrogen and stored at −80°C for DNA extraction. Then, 500 g of samples were stored in a refrigerator at 4°C for the determination of physicochemical parameters. Three replicates of the Xiaoqu sample were crushed and stored in the same manner as the fermented rice sample.

### Physicochemical Factors Determination

The temperature of fermented rice in clay vats was determined by a temperature sensor in real-time. The pH value and acidity were determined by the samples collected from the clay vats. The pH value was measured at room temperature with a pH meter (PHSJ-4F, Leici, Shanghai, China). The acidity was determined as follows: 10 g of the sample was weighed and placed in a mortar and ground thoroughly. Next, 100 mL of distilled water was added, and the mixture was stirred well, allowed to stand for 30 min, filtered through qualitative filter paper, and titrated with NaOH solution (Hu et al., 2021b).

The flavor components were determined by gas chromatography. A 50 mL centrifuge tube was filled with 30 mL of thawed fermented grain sample, sonicated at a low temperature for 10 min, and centrifuged at 10,000 r/min, 4°C for 10 min. Then, 10 mL of the supernatant was pipetted, added to 0.2 mL of n-butyl acetate internal standard, and mixed well. Finally, the mixture was filtered through a 0.22 μM filter membrane to obtain the sample to be tested, which was detected on a GC-2014C Shimadzu gas chromatograph with a mixed standard of baijiu as the reference standard. The detection conditions were as follows: packed column; carrier gas (high-purity nitrogen) flow rate, 180 mL/min; hydrogen flow rate, 40 mL/min; air flow rate, 400 mL/min; detector temperature, 180°C; injector temperature, 180°C; column temperature, 90°C; isothermal.

### DNA Extraction

The samples were weighed (5 g of Xiaoqu; 15 g of fermented rice) and subjected to the following pretreatment: suspension with 30 mL of sterilized 0.1 mol/L PBS buffer, addition of three glass beads, vortex shaken for 5 min, centrifuged at 300 r/min for 5 min to obtain the supernatant; washing of the precipitate with PBS buffer three times, and centrifugation to collect the supernatant. The supernatant was centrifuged at 9,000 r/min for 3 min, the resulting supernatant was discarded, and the cell precipitate was collected. The precipitate was washed three more times with 5 mL of PBS, centrifuged at 9,000 r/min for 3 min each time, and finally resuspended in 2 mL of PBS solution, after which DNA was extracted using the Omega EZNA^TM^ Soil Genome Extraction Kit (Omega, GA, United States) according to the manufacturer’s instructions. The collected DNA sample was quantified, and the mass of the extracted DNA was measured. The sample criteria for sequencing analysis were concentration >50 ng/L and clear bands on the electropherogram.

### High Throughput Sequencing

The purified genomic DNA was sequenced by Shanghai Personal Biotechnology Co., Ltd. (China) using Illumina Miseq PE300. Sequencing was performed on the 16S rRNA gene V3 and V4 regions for bacteria as well as the ITS1 region for fungi (Dorn-In et al., 2013; Los et al., 2020). Bacteria were amplified with primers F338 and R806 (5′-ACTCCTACGGGAGGCAGCAG-3′; 5′-GGACTACHVGGGTWTCTAAT-3′) for 16S rRNA gene library construction. Fungi were amplified with primers ITS5-1737F and ITS2-2043R (5′- GGAAGTAAAAGTCGTAACAAGG-3′; 5′-GCTGCGTTCTTCATCGATGC-3′) for ITS1 gene library construction. The PCR products were purified using the QIAquick PCR Purification Kit (Qiagen, Valencia, CA, United States) validated on a 2% agarose gel and diluted to equal concentrations. Finally, paired-end sequencing was performed on a MiSeq sequencer followed by splicing (Hong et al., 2016). The raw sequencing data were submitted to NCBI under sequence number PRJNA699433.

### Bioinformatics and Statistical Analysis

Raw data obtained from high-throughput sequencing were processed with QIIME2 (2019.4) and subjected to quality control at 99% accuracy to remove duplicate sequences, sequences with short read length, low-abundance sequences, and chimeras. Sequences that remained well above the number in the species after quality screening and de-redundancy were subjected to OTU clustering at 97% similarity to obtain representative sequences and generate a table of OTUs (Li et al., 2011). OTUs were optimized by random sampling and processed by subsampling (sequence number of bacteria: 18,022; sequence number of fungi: 26,084). The OTUs of bacteria and fungi were classified and annotated based on the Silva database (Release 132^1^) and the UNITE database (Release 8.0^2^), respectively. Based on the OTU table, analysis of species composition and diversity indices was performed. Alpha diversity was characterized by Chao1 and observed species indices for richness, Shannon and Simpson indices for diversity, Faith’s PD index for evolutionary-based diversity, and Pielou’s evenness index for evenness. Beta diversity was characterized by non-metric multidimensional scaling (NMDS) and the NMDS model was evaluated by stress values. The R package VennDiagram was used to plot Venn diagrams based on the OTU table, and the number of members in each set was counted separately according to the presence or absence of OTUs among the groups. Redundancy analysis (RDA) was performed on the OTU table of samples after Hellinger pre-transformation, variance inflation factor was used to test for multicollinearity of the explanatory variables, and the reliability of RDA model was assessed by permutation test (*P* < 0.05). NMDS and RDA analyses as well as plotting were performed using the R package vegan (2.5–6).

To compare the microbial communities in the production process of rice-, light-, and strong-flavor baijiu, high-throughput sequencing data from the production process of light-flavor baijiu (PRJNA699760) and strong-flavor baijiu (PRJNA622890) were downloaded from the NCBI database. The sequencing data of the rice-flavor baijiu in this study, and the other two kinds of baijiu were combined and analyzed using NMDS. These sequencing data targeted the same gene regions; V3–V4, for bacteria and ITS1 for fungi.

Alpha diversity values were analyzed using IBM SPSS Statistics 25.0 software. Tukey’s HSD test was performed to identify the statistical significance of differences between treatments, with *P* < 0.05 indicating a significant difference.

## Results

### Changes in Physicochemical Factors

**FIGURE 2:**
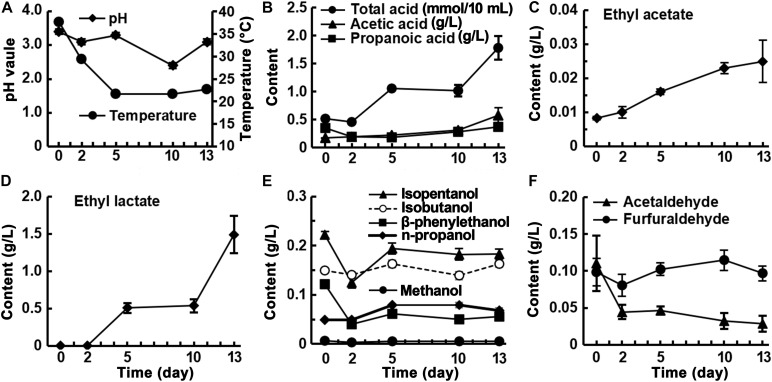
Changes of physicochemical factors in fermented rice during the fermentation of rice-flavor baijiu.

Fermentation began at pH 3.4, which dropped to pH 2.4 after 10 days and rose to pH 3.4 at the end of fermentation (Figure 2A). The trend in total acid did not correspond exactly to the changes in pH, decreasing slightly from day 0 to day 2, then increasing rapidly and stabilizing from day 5 to day 10, and increasing rapidly after 10 days. The acetic acid content showed an increasing trend, eventually reaching 0.58 g/L, and the propionic acid content decreased during the first 2 days of fermentation and then increased to 0.37 g/L at the end. The accumulation rates of acetic and propionic acids accelerated during the final 3 days of fermentation (Figure 2B). The levels of ethyl acetate and ethyl lactate increased as fermentation proceeded (Figures 2C,D). Ethyl lactate was undetectable during the first 2 days and then rapidly increased to 1.49 g/L after 13 days, which was 60 times the amount of ethyl acetate present (Figure 2D). Five alcoholic flavor substances; namely, isoamyl alcohol, isobutanol, β-phenylethanol, n-propanol, and methanol, were detected in the fermented rice samples. The levels of these substances, with relatively high starting concentrations, showed a decreasing trend in the first 2 days of fermentation then increased and stabilized. For example, the initial level of β-phenylethanol, a characteristic component of rice-flavor, was 0.12 g/L, which rapidly decreased to 0.04 g/L in the first 2 days of fermentation and then slowly increased to 0.067 g/L at the end of fermentation (Figure 2E). This indicates that visible β-phenylethanol is mainly produced during the saccharification stage and is partially decomposed during the fermentation stage. The furfural content remained stable and the acetaldehyde content showed a decreasing trend (Figure 2F). The analysis of the volatile components in the fermented grains during fermentation showed that, except for ethyl lactate, which accumulated rapidly and in large quantities during the fermentation process, and acetic acid and ethyl acetate, whose levels increased slowly, the other substances were already produced in the saccharification stage, and the contents did not change substantially before and after fermentation.

### Changes in the Microbial Community

**FIGURE 3:**
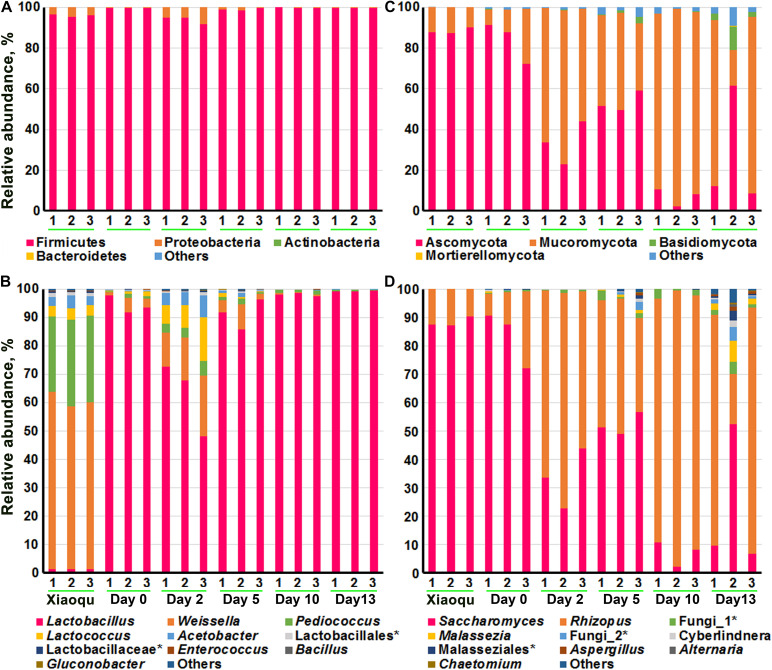
The composition of microbial community in fermented rice in the production of rice-flavor baijiu. Bacteria at phylum **(A)**, and genus **(B)** levels. Fungi at phylum **(C)**, and genus **(D)** levels. 1, 2, and 3 represent three repeats. The rank in genera (top 10) was obtained according to the average relative abundance of all samples.

Figure 3 demonstrated the microbial abundance of bacteria and fungi at the phylum and genus levels. At the phylum level, bacteria mainly consisted of Firmicutes and Proteobacteria, with Firmicutes dominating. The top 10 genera of bacteria obtained according to the average relative abundance of all samples were shown in Figure 3B and Supplementary Table 1, including *Lactobacillus*, *Weissella*, *Pediococcus*, *Lactococcus*, and *Acetobacter*, etc., and the proportion of these five genera in the brewing process was above 97%. The genus *Bacillus* although in the top 10 genera, were present in extremely low proportions. In Xiaoqu, the order of proportions from high to low was *Weissella* (59.53%), *Pediococcus* (29.18%), *Acetobacter* (3.65%), and *Lactobacillus* (1.35%). The species of *Lactobacillus* were dominant during production (62.88–99.23%), with a proportion of 94.25% at the end of saccharification and a significant decrease followed by a significant increase during fermentation. Among the microorganisms that could be identified at the species level, *Lactobacillus helveticus*, *Lactobacillus fermentum*, and *Weissella paramesenteroides* had the highest abundance. The highest abundance of *L. helveticus* was probably from the environment or raw rice material rather than from Xiaoqu (Supplementary Table 2).

At the phylum level, fungi included Ascomycota, Mucoromycota, Basidiomycota, etc., of which the first two were dominant. The top 10 genera of fungi were shown in Figure 3C and Supplementary Table 3, with *Saccharomyces* (7.06–83.50%) and *Rhizopus* (15.21–90.89%) being dominant. For example, *Saccharomyces* constituted 88.41% of fungi genera in Xiaoqu, with a significant decrease in abundance during fermentation. *Rhizopus* was present at 15.21% at the beginning of fermentation and increased significantly during fermentation, reaching up to 90.89% by day 10. In third place was an unclassified fungus, indicating that non-culturable fungi may be involved in the brewing process of rice-flavor baijiu. At the species level, *S. cerevisiae, Rhizopus arrhizus*, and *Rhizopus microsporus* were dominant, and the proportions of all these three microorganisms changed significantly (*P* < 0.5) during the fermentation process, with the overall trend being that *S. cerevisiae* significantly (*P* < 0.5) decreased, *R. arrhizus* and *R. microsporus* significantly increased, and the proportions were 53.53 and 8.21% at the end of fermentation, respectively (Supplementary Table 4).

### Alpha Diversity Analysis

Alpha-diversity was calculated at the OTU level to evaluate the changes in microbial diversity during the production of rice-flavor baijiu (Table 1). During fermentation, the number of bacterial species first increased significantly, was highest on day 2, then decreased significantly. The trends of the Simpson and Shannon indices of bacteria as well as the number of species were the same, and at the end of the process, the species number and diversity indices were the lowest. There were only five fungal species in Xiaoqu, which was far fewer than the number of bacterial species. The number of fungal species did not change significantly from day 0 to day 10 during fermentation and increased significantly at the end. The Simpson and Shannon indices of fungi showed an increasing trend.

**TABLE 1:** Alpha diversity of bacteria and fungi based on high throughput sequencing.

**Sample**	**Xiaoqu**	**Day 0**	**Day 2**	**Day 5**	**Day 10**	**Day 13**	**SEM**	**P-value**
**Bacteria**								
Chao1	262.0^b^	180.6^cd^	345.9^a^	242.3^bc^	150.8^de^	102.8^e^	20.3	<0.001
Observed_species	245.5^b^	169.4^cd^	303.1^a^	224.8^bc^	143.6^de^	96.9^e^	17.5	<0.001
Simpson	0.90^a^	0.82^ab^	0.91^a^	0.79^b^	0.60^c^	0.68^c^	0.03	<0.001
Shannon	4.66^ab^	4.05^b^	4.99^a^	4.21^ab^	3.02^c^	2.77^c^	0.21	<0.001
Faith_pd	3.87^b^	3.04^cd^	4.88^a^	3.58^bc^	2.55^d^	2.69^d^	0.21	<0.001
Pielou_e	0.59^a^	0.55^a^	0.61^a^	0.54^a^	0.42^b^	0.42^b^	0.02	<0.001
**Fungi**								
Chao1	5.0^c^	19.4^bc^	11.3^bc^	30.4^b^	14.7^bc^	81.1^a^	27.9	<0.001
Observed_species	5.0^c^	19.3^bc^	11.3^bc^	30.3^b^	14.6^bc^	80.6^a^	27.8	<0.001
Simpson	0.20^c^	0.27^c^	0.47^b^	0.61^b^	0.48^b^	0.55^b^	0.17	0.00
Shannon	0.52^d^	0.74^d^	1.10^cd^	1.84^b^	1.52^bc^	2.21^b^	0.69	0.00
Faith_pd	2.22^d^	7.16^bc^	5.41^cd^	9.34^b^	5.10^cd^	19.58^a^	5.91	<0.001
Pielou_e	0.21^bc^	0.18^c^	0.32^ab^	0.38^a^	0.40^a^	0.35^a^	0.10	0.00

*SEM (standard error of the mean); *n* = 3; ^*a,b,c,d,e*^Mean values in the same row with different superscripts differ significantly (*P* < 0.05).*

### Beta Diversity Analysis

**FIGURE 4:**
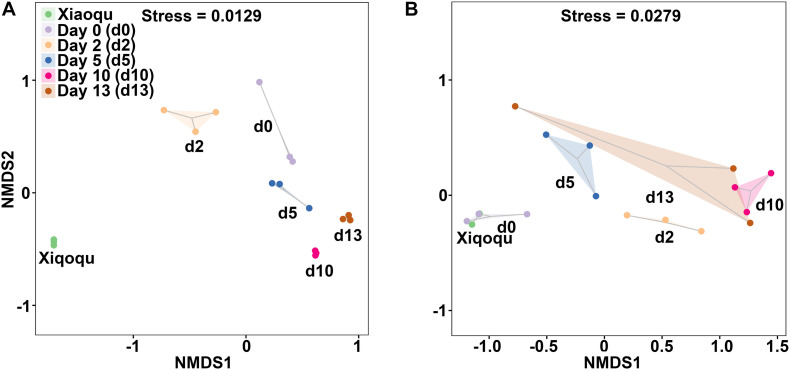
Non-metric multidimensional scaling (NMDS) ranking of microbial community in the production of rice-flavor baijiu. Bacteria **(A)**, and fungi **(B)**. A closer distance between points indicates higher similarity; *n* = 3.

The NMDS analysis based on the Bray–Curtis distance was used to demonstrate beta diversity (Figure 4). In the analysis of bacteria, the three replicates were close to each other and the samples were easily distinguishable among groups, indicating that the bacterial communities were more different among groups. In the analysis of fungi, one sample on day 13 was more variable and similar to the samples on day 5, while the three replicates in the other groups were close to each other. The samples from two groups; Xiaoqu and samples at the end of saccharification, were close to each other, while during fermentation, d0, d2 d5, and d10 were well-distinguished, indicating that the fungal communities differed more among these four groups; the differences in fungal community composition in the fermented grains at the late stage of fermentation (days 10 and 13) were relatively small and the community structure was gradually stabilized.

### Shared Species Analysis

**FIGURE 5:**
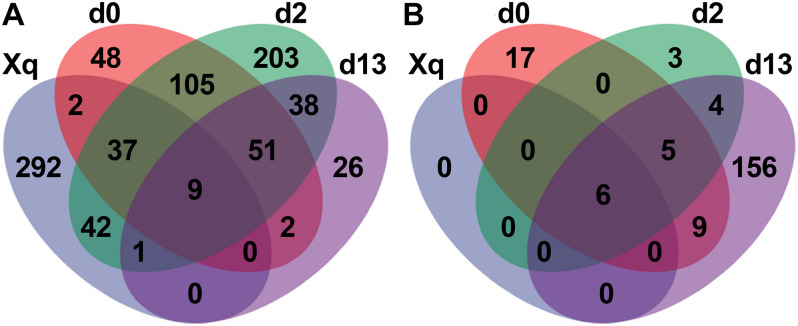
Venn diagram showed the microbial species in Xiaoqu and fermented rice based OTUs obtained by sequencing. Bacteria **(A)**, and fungi **(B)**.

The four groups of samples; Xiaoqu, day 0 (d0), day 2 (d2), and day 13 (d13), were used to construct the Venn diagram (Figure 5). In the bacterial community, 383 species of bacteria were detected in Xiaoqu, of which only 48 species were shared with the d0 sample and 10 with the end of fermentation, indicating that more species of bacteria in the saccharification and fermentation process came from the environment and raw materials, such as rice. As for the fungal community, there were only six species of fungi in Xiaoqu. These six fungal species were shared with d0, d2, and d13 samples. According to Supplementary Table 4, four of these were *S. cerevisiae*, *R. arrhizus*, *R. microsporus*, and *Cyberlindnera fabianii.* These four species of fungi, especially *S. cerevisiae* and *R. arrhizus*, played a dominant role in the brewing process. Moreover, at the end of fermentation, 156 of the detected fungi were not present in Xiaoqu, d0, or d2, indicating that fungi tended to grow in the fermented grains with time.

### Association Between Microbial Community and Physicochemical Factors

**FIGURE 6:**
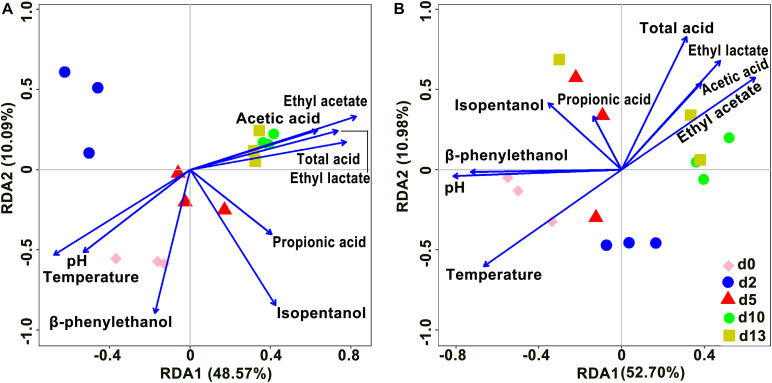
Redundancy analysis (RDA) showed the relationship of microbial community and physicochemical factors in the brewing process of rice-flavor baijiu. Bacteria **(A)**, and fungi **(B)**.

Redundancy analysis was performed to reveal the relationships between the microbial community and physicochemical factors based on OTUs (Figure 6). In the analysis of bacteria, axis 1 and axis 2 explained 48.57 and 10.09% of the total variance, respectively, and in the analysis of fungi, axis 1 and axis 2 explained 52.70 and 10.98% of the total variance, respectively. Among fungi and bacteria, the angles among ethyl lactate, ethyl acetate, total acid, and acetic acid were all acute, indicating a positive correlation among these four variables. Total acid was the main variable affecting the composition of d10 and d13 species; temperature was always negatively correlated with total acid, i.e., total acid always decreased with increasing temperature. As the fermentation time progressed, the species composition of the community gradually changed from being controlled by temperature to being controlled by total acid. Furthermore, in the analysis of bacteria, d0, d2, and d5 species composition were mainly influenced by pH and temperature. In the fungi, ethyl acetate was positively correlated with acetic acid, pH was positively correlated with β-phenylethanol, and temperature was negatively correlated with ethyl acetate, with temperature being the main variable affecting d0 and d2 species composition.

### Comparison of the Microbial Community in Three Different Flavors of Baijiu

**FIGURE 7:**
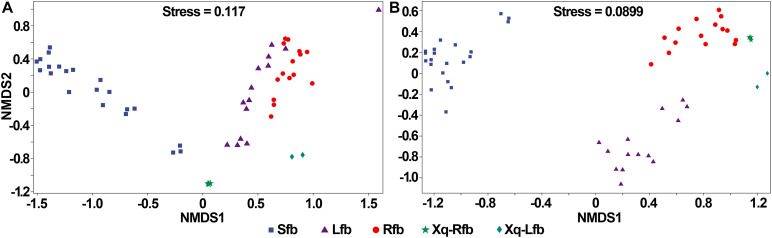
Non-metric multidimensional scaling (NMDS) ranking of microbial community in production of rice-flavor baijiu, light-flavor baijiu, and strong-flavor baijiu. Bacteria **(A)**, and fungi **(B)**. Sfb, strong-flavor baijiu, fermented grains samples were separately collected on days 0, 7, 15, 25, 45, 70, and 95, *n* = 3; Lfb, light-flavor baijiu, fermented grains were separately collected on days 0, 1, 3, 5, 7,10, and 15, *n* = 2; Rfb, rice-flavor baijiu, samples collected in this study. Xq-Rfb, Xiaoqu used in rice-flavor baijiu, *n* = 3; Xq-Lfb, Xiaoqu used in light-flavor baijiu, *n* = 2. Sample information used for bacterial and fungal analyses were shown in Supplementary Tables 5, 6, respectively.

The bacterial and fungal communities in the fermentation process of the three flavor types of baijiu were well distinguished (Figure 7). As shown in Figure 7A, in the analysis of bacteria, the samples were close to each other in the fermentation process of the light-flavor and rice-flavor types, but the two groups could be distinguished from each other; the samples in the fermentation process of the strong-flavor type of baijiu were close to each other and were well-distinguished from the light-flavor and rice-flavor types; the samples of the two starters were easy to reproduce and each was separated from the other groups. In the analysis of fungi, for the fermentation process samples of light-, rice-, and strong-flavor baijiu, each of the three groups clustered together separately (Figure 7B). These results indicate that different flavor types of baijiu possess unique microbial communities.

## Discussion

The flavor components of baijiu are complex, with nearly 100 types in strong- and sauce-flavor baijiu, and more than 50 types in light-flavor baijiu (Dai et al., 2020; Huang et al., 2020). In comparison, the rice-flavor baijiu has fewer flavor components. Only 11 major flavor components were identified in this study, which might be the main reason for the weak aroma of rice-flavor baijiu. Ethyl lactate, ethyl acetate, and β-phenylethanol are the characteristic components of rice-flavor baijiu that establish the style characteristics (Arbab et al., 2020). In this study, the content of ethyl lactate was much higher than that of ethyl acetate and β-phenylethanol, with the former being more than 10 times that of the latter, which is in line with the typical characteristics of rice-flavor baijiu. Ethyl lactate was produced during the fermentation of rice-flavor baijiu, while the other 10 components were already produced at the saccharification stage, and other components, such as β-phenylethanol, had mostly completed accumulation at the saccharification stage.

The participation of multiple microorganisms in the fermentation process is a major feature of baijiu production and an important basis for the complexity of the flavor substances (Zou et al., 2018). In this study, the species diversity of bacteria was much higher than that of fungi, and the bacterial diversity index increased then decreased as fermentation progressed, while the fungal diversity tended to increase. As the production of baijiu moves from the saccharification stage to the fermentation stage, the intact rice grains gradually become crushed as the starch in them is digested, and the water held by the starch is gradually released. Some water is also produced while the sugar is broken down. These actions jointly cause the water content in the fermentation materials to increase, and the fermentation state changes from solid to semi-solid and further to liquid state. The changes in material form lead to changes in the composition and diversity of the microbial community. At the early stage of fermentation, the fermented rice is rich in nutrients and gradually change from an aerobic to an anaerobic environment. After the microorganisms adapt to the environment, those from the raw materials and the environment grow rapidly; thus, diversity increases. At the late stage of fermentation, the anaerobic environment is dominant, the nutrient content decreases, and the acidity and alcoholic content increases, which inhibit the growth of some microorganisms and lead to a decrease in species diversity. The changing pattern of microbial diversity is consistent with that in the brewing of strong- (Tan et al., 2019; Guan et al., 2020), mixed- (Liu and Miao, 2020), and light-flavor baijiu (Dong et al., 2020).

Bacteria are involved in the brewing process of baijiu and influence the production of flavor substances (Arbab et al., 2020). In this study, during the brewing process, *Lactobacillus* (62.88–99.23%) was the dominant genus, followed by *Weissella*, *Pediococcus*, *Lactococcus*, and *Acetobacter*. Among them, the former four are lactic acid bacteria and are the main lactic acid source, while *Acetobacte*r is a strong producer of acetic acid (Wu X. et al., 2017). *Weissella* was the dominant genus in the starter Xiaoqu, and *Lactobacillus* rapidly became the dominant genus during the fermentation stage, with its abundance at the end of fermentation being 99.23%. *Lactobacillus* sp. are more likely to undergo homolactic fermentation, which is the main reason for the rapid increase in ethyl lactate content and only a small increase in acetic acid and ethyl acetate content at the late stage of fermentation. The main bacterial genera in the Xiaoqu light-flavor baijiu were *Lactobacillus*, *Acetobacter*, *Weissella*, *Lactococcus*, and *Bacillus* (Dong et al., 2020; Hu et al., 2021a,b). In strong-flavor baijiu, the main bacteria genera were *Kosakonia*, *Lactobacillus*, *Weissella*, *Pediococcus*, *Pantoea*, and *Bacillus*, which accounted for more than 86% of the bacterial abundance (Guan et al., 2020).

Fungi play a key role in the brewing of baijiu. In this study, the dominant fungi at the genus level were *Saccharomyces* (7.06–83.50%) and *Rhizopus* (15.21–90.89%). From Xiaoqu, at the beginning of fermentation and to the end of fermentation, *Rhizopus* was the dominant flora and the main fungi involved in the brewing of rice-flavor baijiu. Yeast draws sugar substances into the cells and decomposes monosaccharides into carbon dioxide and alcohol under anaerobic conditions (Lin et al., 2017; Wu Q. et al., 2017; Wang J. et al., 2018; Duan et al., 2020). *Rhizopus* and other molds mainly act at the early fermentation stage, providing amylase and glycolase for the fermentation process (Liu et al., 2018), degrading large molecules, such as starch and protein in raw materials, producing small molecules, such as reducing sugars and amino acids for use by yeast, etc., and producing organic acids, aromatic series, etc., which have an important impact on the quality of the liquor (Wang et al., 2008). Generally, *Rhizopus* plays major roles in the saccharification stage, and gradually decline in the fermentation stage due to the decrease in oxygen content, while the abundance of other acid-tolerant and alcoholic anaerobic fungi, such as yeast, gradually increases (Wu H. et al., 2017; Yin et al., 2020a). However, in this study, *Rhizopus* maintained high abundance from the beginning to the end of fermentation, which was very different to the microorganisms in the brewing of light- and strong-flavor baijiu (Guan et al., 2020; Hu et al., 2021b). The main fungi in light-flavor baijiu brewing are *Saccharomyces*, *Aspergillus*, *Rhizopus*, *Pichia*, *Candida*, and *Wickerhamomyces* (Hu et al., 2021b). In the brewing of strong-flavor baijiu, the main fungi are *Kazachstania*, *Thermoascus*, *Aspergillus*, *Saccharomyces*, *Thermomyces*, etc. (Guan et al., 2020).

The NMDS analysis showed that the microbial communities of the three flavor types of baijiu formed their own distinct systems during the fermentation of rice-, light-, and strong-flavor baijiu, and varied greatly among the different flavor types. Compared with the microorganisms of light- and strong-flavor baijiu brewing, the microbial composition of light-flavor baijiu was simple, with few major microbial groups, and with bacteria dominated by *Lactobacillus* and fungi dominated by *Saccharomyces* and *Rhizopus*. In contrast, the other two flavor types of baijiu had many major microbial groups, and in addition to these three microorganisms, *Lactococcus*, *Bacillus*, etc., in bacteria, and a variety of non-*Saccharomyces* yeasts among fungi had high abundance. The small number of microbial species and the prominence of dominant microorganisms may be the main reason for the thin aroma and few flavor substances in rice-flavor baijiu. As a traditional type of baijiu, the production process of rice-flavor baijiu has been passed down between generations (Yin et al., 2020b). The brewing microbial community has been naturally formed through years of domestication, and its simple microbial composition and small number of species means that process improvements can easily be achieved, for example, synthesizing microbial communities by artificial means to replace the traditional Xiaoqu, improving the fermentation method to reduce the interference from undesired microbes, etc.

## Conclusion

In conclusion, this study revealed the microbial composition and diversity in the production of rice-flavor baijiu. The results showed that the species diversity of bacteria was much higher than that of fungi in the brewing process of rice-flavor baijiu, and the bacterial diversity index first increased and then decreased, while the fungal diversity showed an increasing trend. A variety of major microorganisms originated from the environment and raw rice materials. The main bacteria were *Lactobacillus*, and the main fungi were *Saccharomyces* and *Rhizopus*. Temperature and total acid content were the main physicochemical factors affecting the microbial composition. In addition, this study for the first time, to our knowledge, compared the high-throughput sequencing data of microbial communities of three basic types of baijiu; namely, rice-, light-, and strong-flavor types, and showed that the microbial communities differed significantly among the different flavor types of baijiu. Further study is needed to demonstrate what causes the differences in microbial composition. This study provides a basis for the production and process improvement of rice-flavor baijiu, and lays the foundation for studying the nature of baijiu flavor formation and the connection among microorganisms.

## Data Availability Statement

The datasets presented in this study can be found in online repositories. The names of the repository/repositories and accession number(s) can be found below: https://www.ncbi.nlm.nih.gov/, PRJNA699433.

## Author Contributions

YH, XL, XZ, LW, and ZZ analyzed the data and wrote the manuscript. YL and XZ performed the experiments. TG, XY, JT, NP, YL, and SZ contributed to manuscript preparation and experimental design. All authors contributed to manuscript revision and approved the submitted version.

## Conflict of Interest

XZ was employed by Guangdong Deqing Incomparable Health Wine Co., Ltd. The remaining authors declare that the research was conducted in the absence of any commercial or financial relationships that could be construed as a potential conflict of interest.

## Footnotes

1 http://www.arb-silva.de

2 https://unite.ut.ee/
