# Supplementary material for: Characteristics of the Microbial Community in the Production of Chinese Rice-Flavor Baijiu and Comparisons With the Microflora of Other Flavors of Baijiu
Source: Front Microbiol. 2021 Apr 29;12:673670. doi: 10.3389/fmicb.2021.673670 (PMC8116502; doi:10.3389/fmicb.2021.673670)
Supplement: Supplementary file 1 [file Table_1.docx]

**Table S1 The top 10 bacteria at the genus level in production of rice-flavor baijiu (%)**

| **Genus** | **Xiaoqu** | **Day 0** | **Day 2** | **Day 5** | **Day 10** | **Day 13** | **SEM** | ***P* value** |
| --- | --- | --- | --- | --- | --- | --- | --- | --- |
| *Lactobacillus* | 1.35^c^ | 94.25^a^ | 62.88^b^ | 91.26^a^ | 97.96^a^ | 99.23^a^ | 8.55 | <0.001 |
| *Weissella* | 59.53^a^ | 3.16^c^ | 16.14^b^ | 5.12^c^ | 0.62^c^ | 0.90^c^ | 5.12 | 0.014 |
| *Pediococcus* | 29.18^a^ | 0.89^c^ | 3.96^b^ | 1.25^c^ | 1.11^c^ | 0.49^c^ | 2.52 | 0.026 |
| *Lactococcus* | 0.38^b^ | 1.00^b^ | 9.88^a^ | 0.69^b^ | 0.03^b^ | 0.01^b^ | 0.94 | 0.014 |
| *Acetobacter* | 3.65^b^ | 0.26^c^ | 5.64^a^ | 0.90^c^ | 0.07^c^ | 0.11^c^ | 0.54 | 0.004 |
| Unclassified_Lactobacillales | 1.17^a^ | 0.29^bc^ | 0.59^b^ | 0.38^bc^ | 0.11^bc^ | 0.00^c^ | 0.11 | 0.001 |
| Unclassified_Lactobacillaceae | 0.65^a^ | 0.09^bc^ | 0.27^b^ | 0.09^bc^ | 0.00^c^ | 0.00^c^ | 0.06 | 0.007 |
| *Enterococcus* | 0.22^a^ | 0.03^cd^ | 0.15^b^ | 0.02^cd^ | 0.07^c^ | 0.00^d^ | 0.02 | 0.001 |
| *Bacillus* | 0.00 | 0.00 | 0.04 | 0.21 | 0.00 | 0.00 | 0.03 | 0.104 |
| *Gluconobacter* | 0.04^b^ | 0.01^b^ | 0.17^a^ | 0.01^b^ | 0.00^b^ | 0.02^b^ | 0.01 | 0.010 |
| Others | 0.56 | 0.01 | 0.29 | 0.07 | 0.02 | 0.04 | - | - |

n=3; SEM (standard error of the mean); a, b, c Mean values in the same row with different superscripts differ significantly (*P* < 0.05). The rank was obtained according to the average relative abundance of all samples.

**Table S2 The top 10 bacteria at the species level in production of rice-flavor baijiu (%)**

| **Species** | **Xiaoqu** | **Day 0** | **Day 2** | **Day 5** | **Day 10** | **Day 13** | **SEM** | ***P* value** |
| --- | --- | --- | --- | --- | --- | --- | --- | --- |
| *Lactobacillus_helveticus* | 0.00^d^ | 26.97^c^ | 9.36^d^ | 42.76^b^ | 62.32^a^ | 51.80^ab^ | 5.57 | <0.001 |
| *Lactobacillus_fermentum* | 0.05^c^ | 32.42^ab^ | 34.46^a^ | 12.51^bc^ | 1.13^c^ | 7.04^c^ | 3.96 | 0.002 |
| *Weissella_paramesenteroides* | 23.41^a^ | 1.22^c^ | 8.17^b^ | 2.54^c^ | 0.29^c^ | 0.07^c^ | 2.03 | 0.009 |
| *Lactobacillus_pontis* | 0.00^c^ | 0.06^c^ | 1.35^bc^ | 2.33^ab^ | 4.33^a^ | 3.12^ab^ | 0.45 | 0.001 |
| *Lactobacillus_oris* | 0.00^a^ | 0.02^a^ | 0.79^a^ | 0.94^a^ | 0.38^a^ | 0.47^a^ | 0.14 | 0.005 |
| *Lactobacillus_crispatus* | 0.00^c^ | 0.85^a^ | 0.18^c^ | 0.50^b^ | 0.15^c^ | 0.23^bc^ | 0.09 | 0.001 |
| *Lactobacillus_acidophilus* | 0.00^c^ | 0.23^a^ | 0.02^bc^ | 0.15^ab^ | 0.09^bc^ | 0.07^bc^ | 0 | 0.001 |
| *Lactococcus_lactis* | 0.20^a^ | 0.01^c^ | 0.10^b^ | 0.01^c^ | 0.00^c^ | 0.00^c^ | 0 | 0.011 |
| *Lactobacillus_delbrueckii* | 0.00^c^ | 0.03^c^ | 0.08^ab^ | 0.16^a^ | 0.01^c^ | 0.00^c^ | 0 | 0.028 |
| *Enterococcus_faecium* | 0.00^b^ | 0.06^b^ | 0.13^a^ | 0.03^b^ | 0.00^b^ | 0.00^b^ | 0 | 0.015 |
| Others | 76.33 | 38.13 | 45.35 | 38.09 | 31.30 | 37.19 | - | - |

n=3; SEM (standard error of the mean); a, b, c Mean values in the same row with different superscripts differ significantly (*P* < 0.05). The rank was obtained according to the average relative abundance of all samples.

**Table S3 The top 10 fungi at the genus level in production of rice-flavor baijiu (%)**

| **Genus** | **Xiaoqu** | **Day 0** | **Day 2** | **Day 5** | **Day 10** | **Day 13** | **SEM** | ***P* value** |
| --- | --- | --- | --- | --- | --- | --- | --- | --- |
| *Saccharomyces* | 88.41^a^ | 83.50^a^ | 33.40^bc^ | 52.40^b^ | 7.06^d^ | 22.98^cd^ | 7.69 | <0.001 |
| *Rhizopus* | 11.57^c^ | 15.21^c^ | 65.71^ab^ | 41.75^bc^ | 90.89^a^ | 61.84^ab^ | 7.66 | <0.001 |
| Unclassified_Fungi | 0.00^b^ | 0.54^ab^ | 0.73^ab^ | 1.99^ab^ | 1.90^ab^ | 2.42^a^ | 0.3 | 0.001 |
| *Malassezia* | 0.00 | 0.22 | 0.01 | 0.65 | 0.02 | 3.88 | 0.43 | 0.08 |
| Unclassified_Fungi | 0.00 | 0.13 | 0.00 | 1.25 | 0.00 | 2.35 | 0.3 | 0.055 |
| *Cyberlindnera* | 0.02^b^ | 0.23^ab^ | 0.11^b^ | 0.52^ab^ | 0.09^b^ | 1.20^a^ | 0.19 | 0.017 |
| Unidentified Malasseziales | 0.00 | 0.09 | 0.02 | 0.45 | 0.02 | 1.43 | 0.14 | 0.089 |
| *Aspergillus* | 0.00^b^ | 0.00^b^ | 0.00^b^ | 0.34^b^ | 0.01^b^ | 0.82^a^ | 0.09 | 0.038 |
| *Alternaria* | 0.00 | 0.01 | 0.01 | 0.07 | 0.00 | 0.43 | 0.05 | 0.098 |
| *Chaetomium* | 0.00 | 0.01 | 0.00 | 0.06 | 0.00 | 0.35 | 0.05 | 0.141 |
| Others | 0.00 | 0.07 | 0.01 | 0.52 | 0.01 | 2.31 | - | - |

n=3; SEM (standard error of the mean); a, b, c Mean values in the same row with different superscripts differ significantly (*P* < 0.05). The rank was obtained according to the average relative abundance of all samples.

**Table S4 The top 10 fungi at the species level in production of rice-flavor baijiu (%)**

| **Species** | **Xiaoqu** | **Day 0** | **Day 2** | **Day 5** | **Day 10** | **Day 13** | **SEM** | ***P* value** |
| --- | --- | --- | --- | --- | --- | --- | --- | --- |
| *Saccharomyces_cerevisiae* | 88.39^a^ | 83.44^a^ | 33.37^bc^ | 52.31^b^ | 7.02^d^ | 22.69^cd^ | 7.69 | <0.001 |
| *Rhizopus_arrhizus* | 11.52^d^ | 15.18^d^ | 65.46^b^ | 30.88^cd^ | 73.91^b^ | 53.53^bc^ | 6.65 | <0.001 |
| *Rhizopus_microsporus* | 0.04^b^ | 0.07^b^ | 0.40^b^ | 10.86^ab^ | 17.00^a^ | 8.21^ab^ | 1.92 | 0.006 |
| *Malassezia_restricta* | 0.00 | 0.21 | 0.01 | 0.62 | 0.02 | 3.75 | 0.41 | 0.076 |
| *Cyberlindnera_fabianii* | 0.01^b^ | 0.22^b^ | 0.11^b^ | 0.52^ab^ | 0.08^b^ | 1.21^a^ | 0.14 | 0.017 |
| *Aspergillus_flavus* | 0.00 | 0.00 | 0.00 | 0.08 | 0.00 | 0.33 | 0.05 | 0.167 |
| *Aspergillus_pseudodeflectus* | 0.00 | 0.00 | 0.00 | 0.12 | 0.00 | 0.12 | 0.02 | 0.061 |
| *Aspergillus_niger* | 0.00 | 0.00 | 0.00 | 0.06 | 0.00 | 0.10 | 0.01 | 0.057 |
| *Thermoascus_aurantiacus* | 0.00 | 0.00 | 0.00 | 0.00 | 0.00 | 0.15 | 0.02 | 0.246 |
| *Aspergillus_penicillioides* | 0.00 | 0.00 | 0.00 | 00.00 | 0.00 | 0.14 | 0.02 | 0.155 |
| Others | 0.05 | 0.87 | 0.65 | 4.54 | 2.01 | 9.76 | - | - |

n=3; SEM (standard error of the mean); a, b, c Mean values in the same row with different superscripts differ significantly (*P* < 0.05). The rank was obtained according to the average relative abundance of all samples.
